# A change in the aggregation pathway of bovine serum albumin in the presence of arginine and its derivatives

**DOI:** 10.1038/s41598-017-04409-x

**Published:** 2017-06-21

**Authors:** Vera A. Borzova, Kira A. Markossian, Sergey Yu. Kleymenov, Boris I. Kurganov

**Affiliations:** 1 Federal State Institution “Federal Research Centre “Fundamentals of Biotechnology” of the Russian Academy of Sciences”, Leninsky pr. 33, Moscow, 119071 Russia; 2 Kol’tsov Institute of Developmental Biology, Russian Academy of Sciences, 26 Vavilova str, Moscow, 119991 Russia

## Abstract

Chemical chaperones including arginine and its derivatives are widely used by biochemists working on the design of agents, which are able to efficiently suppress protein aggregation. To elucidate the mechanisms of anti-aggregation activity of chemical chaperones, methods based on registration of the increment in light scattering intensity must be supplemented with methods for direct detection of the portion of aggregated protein (γ_agg_). For this purpose asymmetric flow field-flow fractionation was used in the present work. It was shown that heat-induced aggregation of bovine serum albumin (BSA) followed the kinetics of the reaction of the second order (0.1 M sodium phosphate buffer, pH 7.0, 70 °C). It was proposed to use *R*_h_ *vs* γ_agg_ plots to characterize the aggregation pathway (*R*_h_ is the hydrodynamic radius of the protein aggregates, which was calculated from the dynamic light scattering data). The changes in the shape of *R*_h_ *vs* γ_agg_ plots in the presence of arginine, arginine amide and arginine ethyl ester are indicative of the changes in the aggregation pathway of BSA aggregation. A conclusion has been made that larger aggregates are formed in the presence of arginine hydrochloride and its derivatives.

## Introduction

Aggregation of non-native unfolded proteins occurs mainly as a result of interaction of exposed hydrophobic protein surfaces and may be prevented by various low-molecular-weight soluble additives^1–6^. Among many low-molecular-weight compounds, arginine (Arg) is one of the additives, which enhance refolding of proteins, suppress aggregation and increase solubility of proteins; the role of Arg in protein–protein interactions has been reviewed by many authors^7–25^.

Results of quantum chemical calculations show that the guanidinium group of Arg interacts with a variety of amino acids^23^. The guanidinium group of Arg forms strong salt bridges with acidic residues of the protein molecule. Such interactions promote protein aggregation due to suppression of electrostatic repulsion as salt effect^26, 27^. It has been shown that Arg molecules in aqueous solutions are able to interact by head-to-tail hydrogen bonding and by accumulation in stacks of guanidinium groups and thus form clusters^28–30^. The methylene groups in the side chains of free Arg and Arg molecules in clusters can mask exposed hydrophobic patches on the surface of protein molecules resulting in suppression of protein aggregation^31, 32^. Das *et al*.^31^ suggested that typically 8–10 molecules are present in an Arg cluster.

When studying effects of Arg and its derivatives on thermal inactivation and aggregation of lysozyme (98 °C, pH 7.1), Shiraki and coworkers^33, 34^ showed that arginine ethylester (ArgEE) and arginine amide (ArgAd) revealed markedly higher protective action than Arg. It is evident that suppression of lysozyme aggregation is mainly due to additive-induced protein stabilization. The higher protective effect of ArgAd and ArgEE in comparison with that for Arg was also observed in the experiments on dithiothreitol (DTT)-induced aggregation of BSA (45 °C, pH 7.0)^35^. The rate-limiting stage for this test system is that of unfolding of the protein molecule (the order of aggregation with respect to protein is equal to unity). Therefore the observed effects of the agents under study on DTT-induced aggregation of BSA are connected with their action on the unfolding stage, but not on the stage of aggregation of unfolded protein molecules. To elucidate the mechanisms of suppression of protein aggregation by Arg and its derivatives, we should use test systems, where the rate-limiting stage is that of aggregation of unfolded protein molecules^36^. Such systems allow estimating direct effect of chemical chaperones on the aggregation stage.

In this work we studied the effect of Arg and its derivatives on the kinetics of thermal aggregation of BSA at 70 °C using dynamic light scattering (DLS) and asymmetric flow field-flow fractionation (AF4). Aggregation of BSA at this temperature follows the kinetics of the reaction of the second order, suggesting that the rate-limiting stage of the overall aggregation process is that of aggregation of denatured protein molecules. It was demonstrated that in the presence of Arg and its derivatives a change in the aggregation pathway for BSA aggregation took place. To characterize the aggregation pathway, *R*_h_ *vs* γ_agg_ plots were used (*R*_h_ is the hydrodynamic radius of protein aggregates and γ_agg_ is the portion of the aggregated protein). The obtained results showed that Arg and its derivatives induced the formation of larger aggregates in the course of thermal aggregation of BSA.

## Results

### Kinetics of BSA aggregation registered by AF4

**Figure 1:**
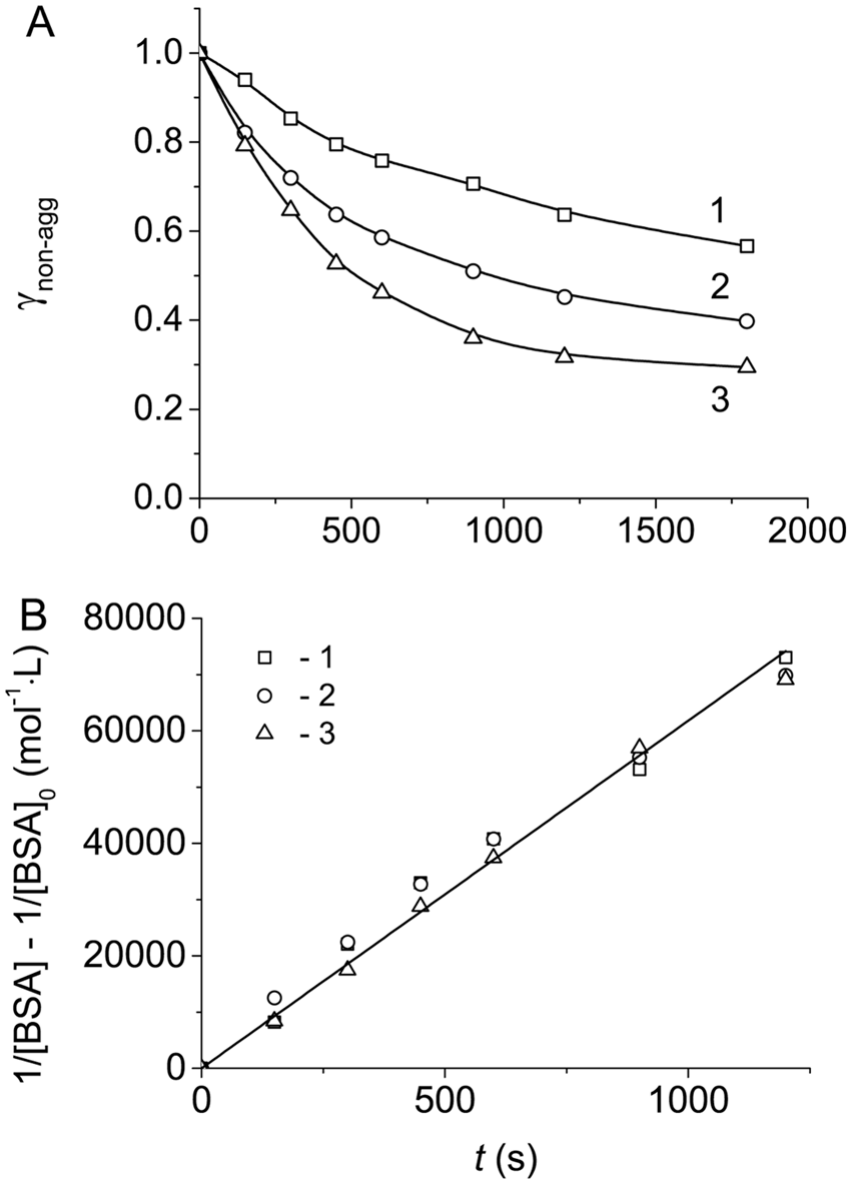
Kinetics of BSA aggregation registered by AF4 (0.1 M Na-phosphate buffer, pH 7.0, 70 °C). (**A**) The dependences of the portion of non-aggregated protein (γ_non-agg_) on time obtained at the following concentrations of BSA: 1 (1), 1.5 (2) and 1.75 mg∙mL^−1^ (3). (**B**) The linear anamorphosis constructed in the coordinates {(1/[BSA] − 1/[BSA]_0_); *t*}.

The kinetics of heat-induced aggregation of BSA at 70 °C in 0.1 M Na-phosphate buffer, pH 7.0, was studied using AF4. Figure 1A shows the dependences of the portion of non-aggregated protein (γ_non-agg_) estimated by AF4 on time. The kinetic curves were obtained at various concentrations of BSA (0.5, 1.0 and 2.0 mg∙mL^−1^). The kinetic data were represented in the coordinates {1/[BSA] − 1/[BSA]_0_; *t*} corresponding to the kinetics of the reaction of the second order^37^:

1 $$\frac{1}{[\mathrm{BSA}]}-\frac{1}{{[\mathrm{BSA}]}_{0}}={k}_{{\rm{II}}}t,$$

2 $${{\rm{\gamma }}}_{\mathrm{non}-\mathrm{agg}}=\frac{[{\rm{BSA}}]}{{[{\rm{BSA}}]}_{0}}=\frac{1}{1+{k}_{{\rm{II}}}{[{\rm{BSA}}]}_{0}t}.$$

In this equation [BSA]_0_ and [BSA] are the initial and current concentration of BSA, respectively, and *k*_II_ is the rate constant of the second order. Equation (2) describes the time-dependent decrease in the portion of non-aggregated protein. The aggregation process is considered as an irreversible transformation of native (non-aggregated) protein into aggregated form. Thus, the protein is represented by two forms, namely by non-aggregated and aggregated proteins. The portion of aggregated protein (γ_agg_) is calculated as (1 − γ_non-agg_):

3 $${{\rm{\gamma }}}_{{\rm{agg}}}=1-{{\rm{\gamma }}}_{\mathrm{non}-\mathrm{agg}}=\frac{{k}_{{\rm{II}}}{[{\rm{BSA}}]}_{0}t}{1+{k}_{{\rm{II}}}{[{\rm{BSA}}]}_{0}t}$$

Linearization of the kinetic data in the coordinates {1/[BSA] − 1/[BSA]_0_; *t*} (Fig. 1B) testifies that heat-induced aggregation of BSA follows the kinetics of the reaction of the second order. The value of *k*_II_ was found to be 62 ± 2 mol·L^−1^·s^−1^. Thus, in the studied range of protein concentrations (0.5–2 mg∙mL^−1^) the initial rate of aggregation (*v*_0_) is proportional to the initial concentration of BSA squared:

4 $${v}_{0}=-{\{\frac{{\rm{d}}[{\rm{BSA}}]}{{\rm{d}}t}\}}_{t\to 0}={k}_{{\rm{II}}}{[{\rm{BSA}}]}_{0}^{2}.$$

### Kinetics of BSA aggregation registered by DLS

To control the kinetics of protein aggregation, methods based on the registration of the increment in the light scattering intensity are widely used. Therefore it was of interest to compare the kinetics of heat-induced aggregation of BSA registered by direct method (AF4) and a method where the increase in the light scattering intensity in time was detected.

**Figure 2:**
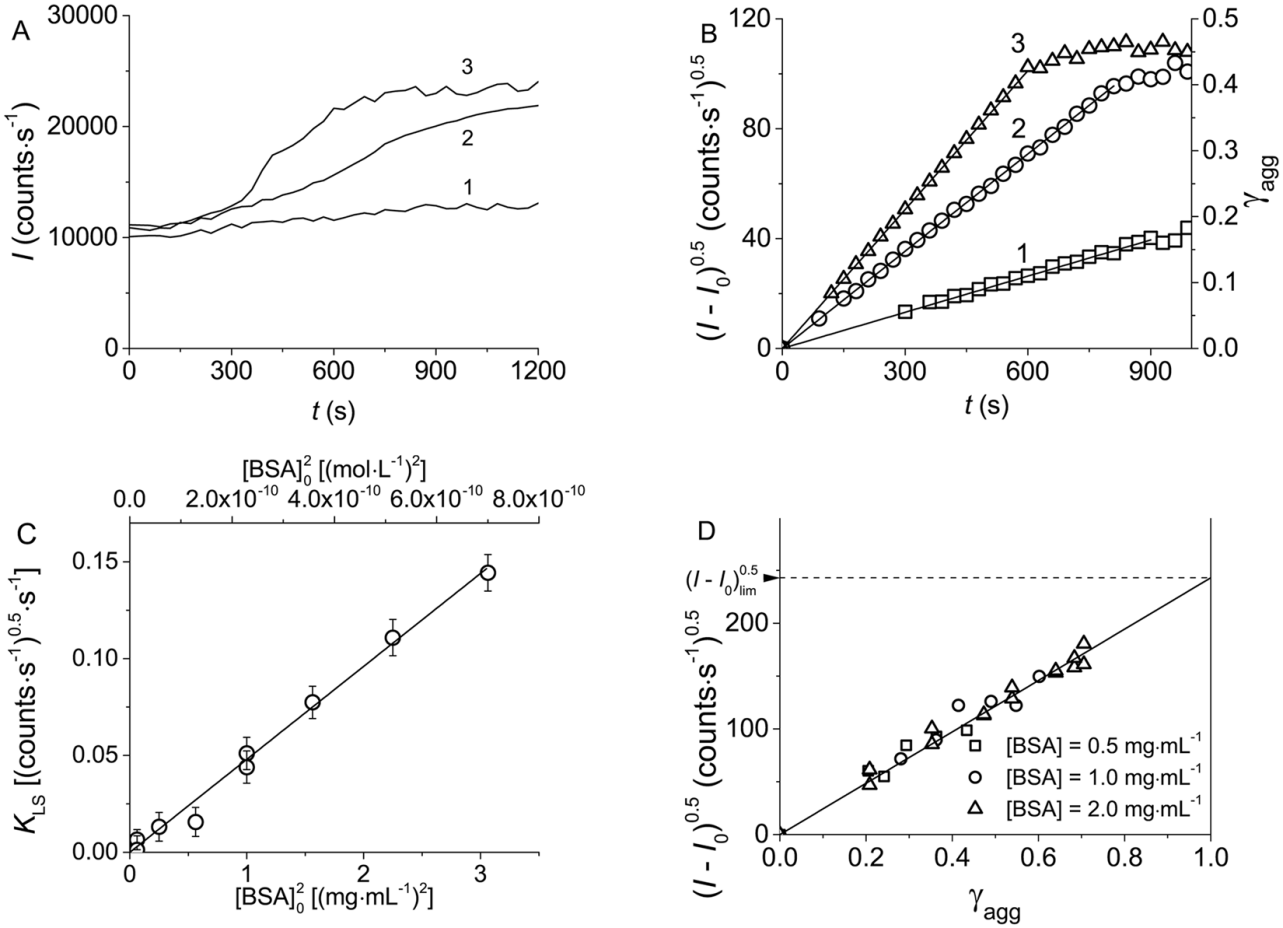
Kinetics of BSA aggregation registered by DLS (0.1 M Na-phosphate buffer, pH 7.0, 70 °C). (**A**) The dependences of the light scattering intensity (*I*) on time obtained at the following concentrations of BSA: 1.00 (1), 1.50 (2) and 1.75 mg∙mL^−1^ (3). (**B**) The kinetic curves represented in the coordinates {(*I* – *I*_0_)^0.5^; *t*} in accordance with Equation (5). The BSA concentrations are the same as on panel A. The γ_agg_ values for the right ordinate axis were calculated from Equation (6). (C) The (*I* − *I*_0_)^0.5^ *vs* the portion of the aggregated protein (γ_agg_) plot constructed at the following concentrations of BSA: 0.5 (1), 1.0 (2) and 2.0 mg∙mL^−1^ (3). (**D**) Parameter *K*_LS_ as a function of ${[{\rm{BSA}}]}_{{\rm{0}}}^{{\rm{2}}}$.

The kinetic curves of thermal aggregation of BSA obtained by DLS at various concentrations of the protein are represented in Fig. 2A. Linearization of the initial parts of the kinetic curves in the coordinates {(*I* − *I*_0_)^0.5^; *t*} (Fig. 2B) indicates that there exists a proportionality between the (*I* − *I*_0_)^0.5^ value and time:

5 $${(I-{I}_{0})}^{0.5}={K}_{{\rm{LS}}}t,$$

where *K*_LS_ is a constant (such a designation for this constant was used in our previous works^35, 38, 39^).

To find a relationship between the increment in the light scattering intensity in the course of BSA aggregation and the portion of aggregated protein (γ_agg_), we have constructed the (*I* − *I*_0_)^0.5^ *vs* γ_agg_ plot. As it is can be seen from Fig. 2D, the relationship between the (*I* − *I*_0_)^0.5^ and γ_agg_ values is linear (*R* ^2^ = 0.9862). This result is in agreement with the data obtained by us earlier (thermal aggregation of BSA was studied in the temperature interval from 60 °C to 80 °C^38^). The dependence of (*I* − *I*_0_)^0.5^ and γ_agg_ is described by the following equation:

6 $${(I-{I}_{0})}^{0.5}={(I-{I}_{0})}_{\mathrm{lim}}^{0.5}\cdot {{\rm{\gamma }}}_{{\rm{agg}}}.$$

The coefficient of proportionality ${(I-{I}_{0})}_{\mathrm{lim}}^{0.5}$ corresponds to the value of (*I* − *I*_0_)^0.5^ at γ_agg_ = 1: ${(I-{I}_{0})}_{\mathrm{lim}}^{0.5}$ = 243 ± 3 (counts/s)^0.5^. The γ_agg_ values on the ordinate axis in Fig. 2B were calculated using Equation (6).

Differentiation of Equation (6) with respect to time gives the following expression:

7 $$\frac{{\rm{d}}{(I-{I}_{0})}^{0.5}}{{\rm{d}}t}={(I-{I}_{0})}_{\mathrm{lim}}^{0.5}\cdot \frac{{d{\rm{\gamma }}}_{{\rm{agg}}}}{{\rm{d}}t}.$$

For the initial parts of the kinetic curves this expression acquires the following form:

8 $${K}_{{\rm{LS}}}=\frac{{(I-{I}_{0})}_{\mathrm{lim}}^{0.5}}{{[{\rm{BSA}}]}_{0}}{v}_{0}={(I-{I}_{0})}_{\mathrm{lim}}^{0.5}{[{\rm{BSA}}]}_{0}{k}_{{\rm{II}}}.$$

When deriving this relationship between the *K*_LS_ and *v*_0_ values, Equations (3) and (4) were taken into account. Thus, parameter *K*_LS_ can serve as a measure of the initial rate of the aggregation process. As expected (see Equation (4)), parameter *K*_LS_ is a linear function of the initial protein concentration squared (Fig. 2D).

### Effects of Arg and its derivatives on the kinetics of BSA aggregation

**Figure 3:**
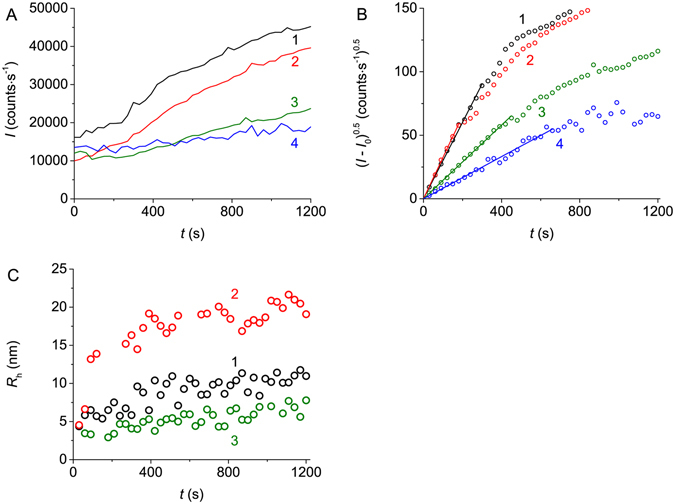
Effect of Arg on the kinetics of aggregation of BSA (1 mg∙mL^−1^) registered by DLS (0.1 M Na-phosphate buffer, pH 7.0, 70 °C). (**A**) The dependences of the light scattering intensity (*I*) on time obtained at the following concentrations of Arg: 0 (1), 50 (2), 350 (3) and 1000 mM (4). (**B**) The kinetic curves represented in the coordinates {(*I* − *I*_0_)^0.5^; *t*}. Concentrations of Arg: 0 (1), 200 (2), 350 (3) and 1000 mM (4). (**C**) The dependences of the hydrodynamic radius (*R*_h_) of protein aggregates on time obtained at the following concentrations of Arg: 0 (1), 200 (2) and 1000 mM (3).

The effects of Arg, ArgAd and ArgEE on the kinetics of BSA aggregation at 70 °C were studied using DLS and AF4. Figure 3 shows the effect of Arg on BSA aggregation registered by DLS ([BSA] = 1 mg∙mL^−1^). The dependences of the light scattering intensity on time obtained at various concentrations of Arg are represented in Fig. 3A. To estimate parameter *K*_LS_, (*I* − *I*_0_)^0.5^ *vs* time plots were constructed (Fig. 3B). The slope of the initial linear parts gives the value of parameter *K*_LS_. The use of DLS allows us to obtain the dependences of the hydrodynamic radius (*R*_h_) of protein aggregates on time (Fig. 3C). The experimental data demonstrating the effects of ArgAd and ArgEE on BSA aggregation are represented in Figs S1 and S2, respectively (Supplementary Information; section S1).

**Figure 4:**
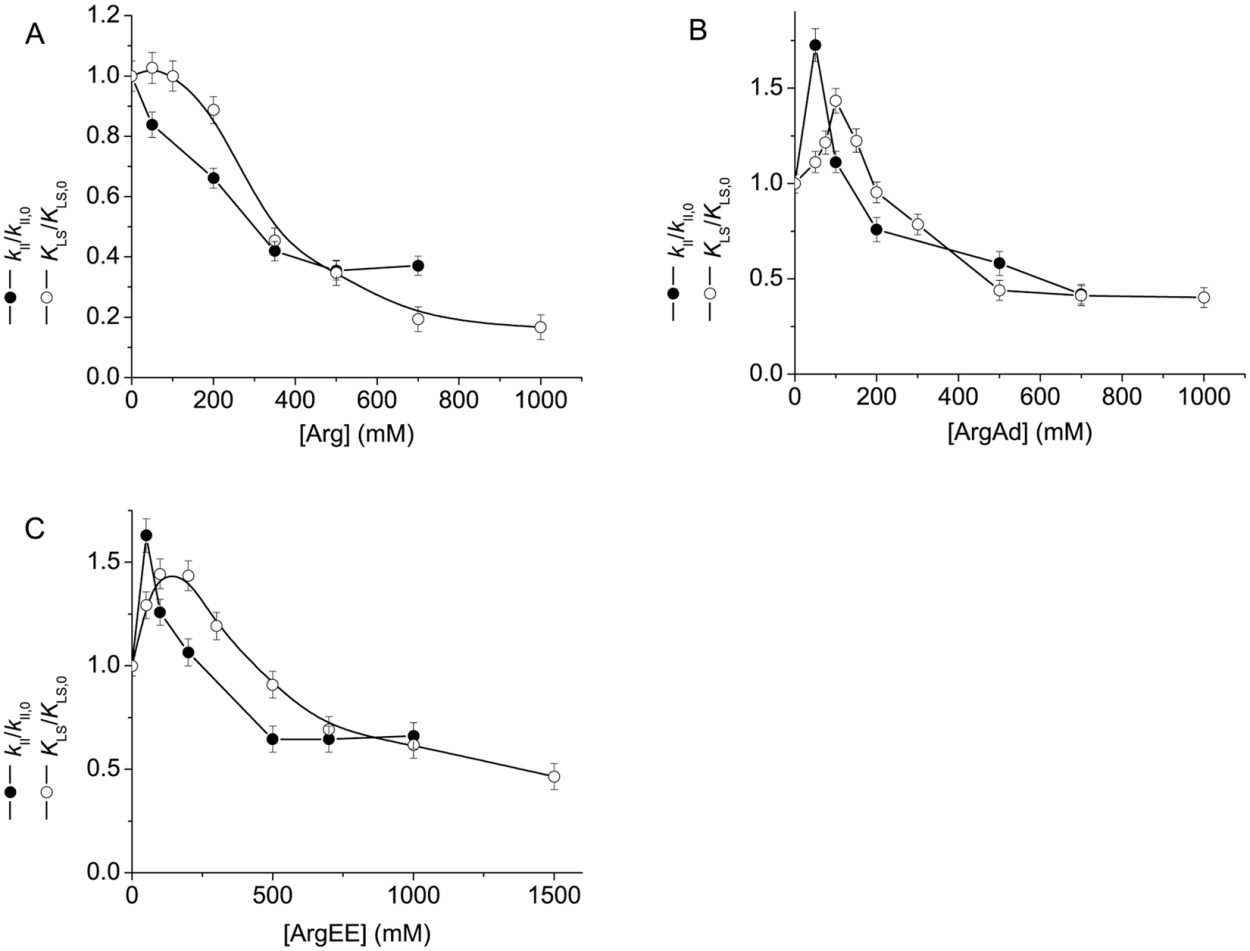
The dependences of the *K*_LS_/*K*_LS,0_ ratio and *k*_II_/*k*_II,0_ ratio on the concentration of Arg (**A**), ArgAd (**B**) and ArgEE (**C**). The error bars were calculated using three independent measurements.

Figure 4 shows the dependences of the *K*_LS_/*K*_LS,0_ ratio on the concentrations of Arg, ArgAd and ArgEE (*K*_LS,0_ is the value of *K*_LS_ in the absence of chemical chaperone). In the case of ArgAd and ArgEE (Fig. 4B and C) the dependence of *K*_LS_/*K*_LS,0_ on the concentration of chemical chaperone passes through a maximum at [ArgAd] ≈100 mM or [ArgEE] ≈200 mM. As for Arg, there is no obvious maximum on the dependence of *K*_LS_/*K*_LS,0_ on Arg concentration (Fig. 4A).

**Figure 5:**
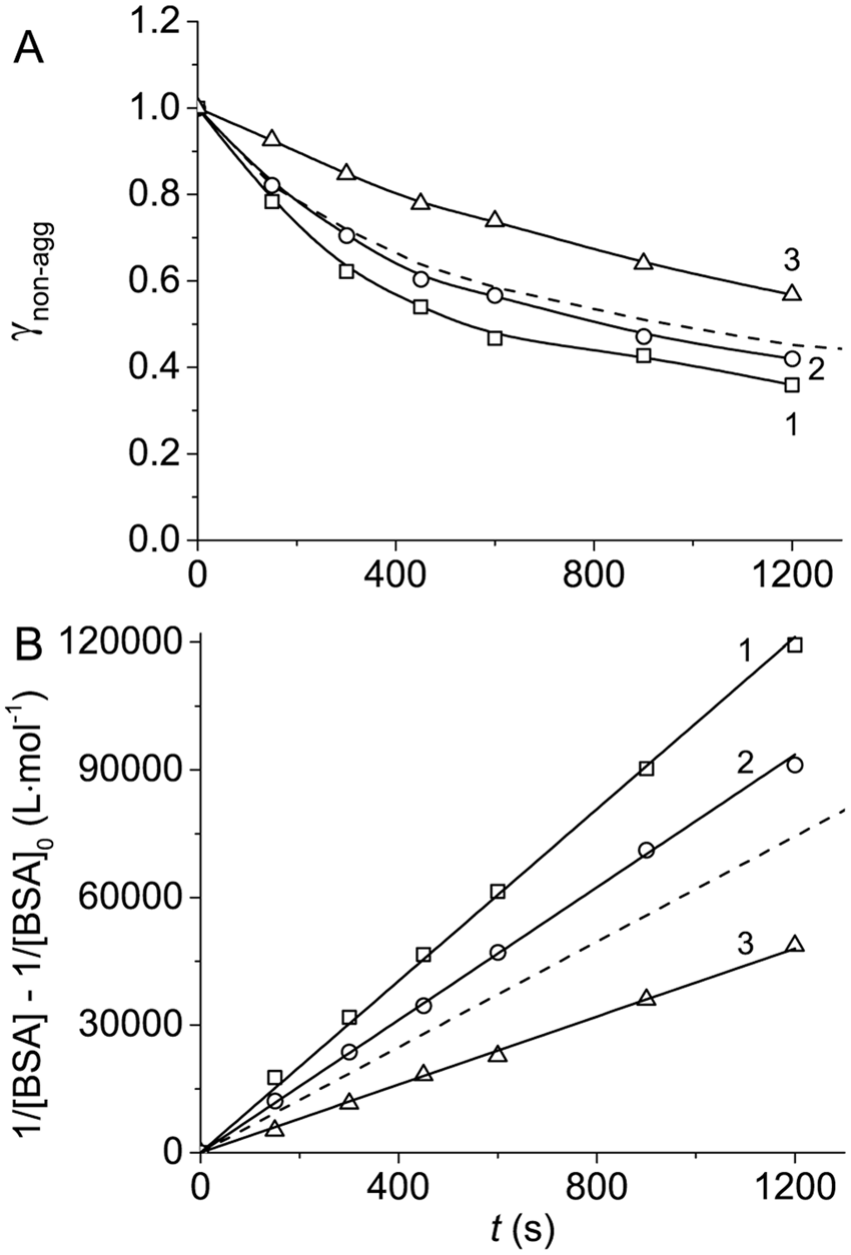
Kinetics of BSA aggregation in the presence of ArgEE registered by AF4. The dependences of the portion of non-aggregated protein (γ_non-agg_) on time (**A**) and the linear anamorphoses constructed in the coordinates {(1/[BSA] − 1/[BSA]_0_); *t*} (**B**) at the following concentrations of ArgEE: 50 (1), 100 (2) and 700 mM (3). The dotted lines on panels A and B correspond to the data obtained in the absence of chemical chaperones.

To correlate the kinetic data obtained by DLS with the direct estimates of the amount of the aggregated protein, we studied the kinetics of BSA aggregation in the presence of Arg and its derivatives using AF4. By way of example, Fig. 5A shows the data on the effect of ArgEE on BSA aggregation kinetics obtained by AF4. The construction of (1/[BSA] − 1/[BSA]_0_) *vs* time plots (Fig. 5B) allowed us to calculate the rate constants of the second order (*k*_II_) at various concentrations of ArgEE. Analogously, the values of *k*_II_ in the presence of different concentrations of Arg and ArgAd were calculated.

The dependences of the relative values of the rate constant of the second order *k*_II_/*k*_II,0_ (*k*_II,0_ is the *k*_II_ value in the absence of chemical chaperone) on the concentration of Arg, ArgAd and ArgEE are represented in Fig. 4, where the values of *K*_LS_/*K*_LS,0_ ratio were also plotted. The fact that the *k*_II_ values determined in the presence of 50 and 100 mM ArgAd or 50 and 100 mM ArgEE exceed the corresponding values obtained in the absence of ArgAd or ArgEE is indicative of the acceleration of the aggregation process at low concentrations of these agents. It is important to note that there are discrepancies between the dependences of *K*_LS_/*K*_LS,0_ on Arg, ArgAd and ArgEE concentrations, on the one hand, and the corresponding dependences for *k*_II_/*k*_II,0_ ratio, on the other hand. By this is meant that, strictly speaking, in the presence of chemical chaperones the *K*_LS_ values cannot be used for the quantitative estimation of the initial rate of aggregation.

Since the addition of Arg and its derivatives to the buffer solution results in the increase in the ionic strength of the medium, we studied the effect of ionic strength on the kinetics of BSA aggregation (see Supplementary Information; section S2). It was shown that the values of parameter *K*_LS_ which is used for characterization of the initial rate of aggregation remained practically unchanged in the interval of NaCl concentration from 0 to 0.5 M.

### The change in the aggregation pathway of BSA in the presence of Arg and its derivatives

A large body of data shows that the change in the environmental conditions or the addition of different agents (osmolytes, small heat shock proteins, denaturants and others) can result in the change in the pathways leading to the formation of protein aggregates of different types^40–43^. It is evident that the data on the size (*R*_h_) of protein aggregates being formed in the aggregation process in conjunction with the direct data on the accumulation of the aggregated protein (γ_agg_) are essential for characterization of the aggregation pathway. If the character of the dependence of *R*_h_ on γ_agg_ remains unchanged at varying the conditions of the experiment (for example, at varying the initial protein concentration or in the presence of any additives), this means that the process of protein aggregation proceeds through the same aggregate states. In this case we can say about the constancy of the aggregation pathway. However, the divergence of the dependences of *R*_h_ on γ_agg_ obtained at various initial protein concentrations or in the presence of any additives is indicative of the change in the aggregation pathway. For example, if at any fixed value of γ_agg_ the higher values of *R*_h_ are observed, this means that the process of protein aggregation proceeds through the formation of larger aggregates.

**Figure 6:**
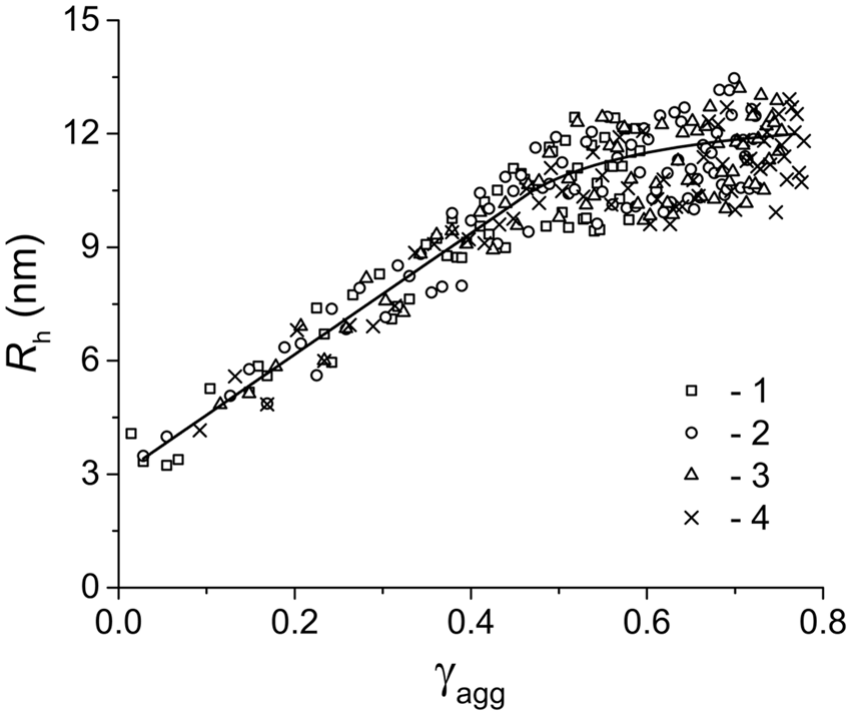
The dependences of the hydrodynamic radius (*R*_h_) of BSA aggregates on the portion of aggregated protein (γ_agg_) calculated at different initial concentrations of the protein: 0.50 (1), 1.00 (2), 1.50 (3) and 1.75 mg∙mL^−1^ (4).

Consider the application of *R*_h_ *vs* γ_agg_ plots for the comparison of the aggregation pathways realized for BSA aggregation in the absence or presence of chemical chaperones. The relationships between the hydrodynamic radius of protein aggregates and the portion of the aggregated protein obtained at various initial concentrations of BSA are represented in Fig. 6. As it can be seen from this Figure, all the experimental points fall on the common curve. This result indicates that the aggregation pathway remains unchanged as the protein concentration is varied.

**Figure 7:**
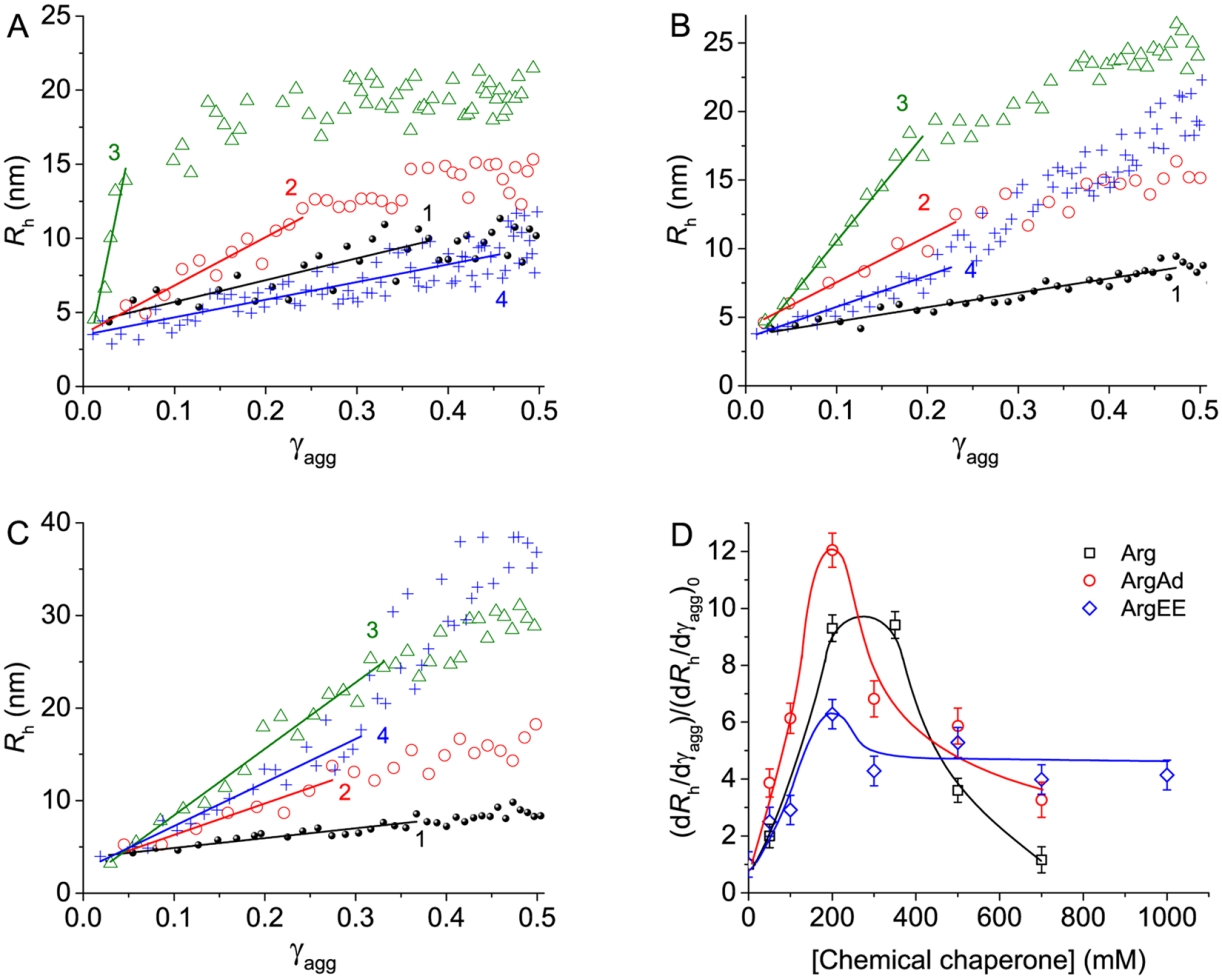
The change in the aggregation pathway for BSA aggregation in the presence of Arg and its derivatives. (**A**) The dependences of the hydrodynamic radius (*R*_h_) of BSA aggregates on the portion of the aggregated protein (γ_agg_) calculated at different concentrations of Arg: 0 (1), 50 (2), 350 (3) and 700 mM (4). (**B**) The dependences of *R*_h_ of BSA aggregates on γ_agg_ calculated at different concentrations of ArgAd: 0 (1), 50 (2), 200 (3) and 700 mM (4). (**C**) The dependences of *R*_h_ of BSA aggregates on γ_agg_ calculated at different concentrations of ArgEE: 0 (1), 50 (2), 200 (3) and 1000 mM (4). (**D**) The dependences of the relative values of the initial slopes (d*R*_h_/dγ_agg_)/(d*R*_h_/dγ_agg_)_0_ on the concentration of Arg, ArgAd and ArgEE. (d*R*_h_/dγ_agg_)_0_ and (d*R*_h_/dγ_agg_) are the initial slopes of the dependences of *R*_h_ on γ_agg_ calculated in the absence or in the presence of additives, respectively. Three independent measurements were used to determine the error bars shown in this figure.

When studying the effect of Arg and its derivatives on the kinetics of BSA aggregation, we observed that the character of the dependence of *R*_h_ on γ_agg_ was changed at varying the concentration of Arg, ArgAd or ArgEE (Fig. 7A,B and C). To characterize the differences between the dependences of *R*_h_ on γ_agg_ obtained at various concentrations of Arg, ArgAd or ArgEE, we calculated the slope of the initial parts of these dependences (d*R*_h_/dγ_agg_). The relative values of this derivative (d*R*_h_/dγ_agg_)/(d*R*_h_/dγ_agg_)_0_ as a function of the concentration of each chemical chaperone are represented in Fig. 7D ((d*R*_h_/dγ_agg_)_0_ is the value of (d*R*_h_/dγ_agg_) in the absence of chemical chaperone). As it can be seen from this Figure, there is an increase in the (d*R*_h_/dγ_agg_)/(d*R*_h_/dγ_agg_)_0_ value in the interval of the chemical chaperone concentration from zero to 200 mM. Further increase in the chemical chaperone concentration results in the diminishing of (d*R*_h_/dγ_agg_)/(d*R*_h_/dγ_agg_)_0_ value, this value remaining higher than unity. Thus, the addition of Arg, ArgAd or ArgEE causes the change in the aggregation pathway for thermal aggregation of BSA at 70 °C. The larger aggregates are formed in the presence of these chemical chaperones.

### Measurements of zeta-potential of BSA aggregates

The measurements of zeta-potential of protein particles give valuable information on their ability to aggregation^44^. To characterize the influence of Arg and its derivatives on assembling of BSA aggregates formed in the course of thermal aggregation, we measured the values of zeta-potential of BSA preparations preheated for 15 min at 70 °C. Zeta-potential for BSA aggregates formed in the absence of any additives was found to be –7.5 ± 0.1 mV. When preheating of BSA was performed in the presence of 200 mM Arg, ArgAd or ArgEE, the following values of zeta-potential were obtained: −4.5 ± 0.1, −2.9 ± 0.1 and −0.5 ± 0.1 mV, respectively.

## Discussion

The results obtained in the present work indicate that in the case of BSA aggregation at 70 °C the order of aggregation with respect to the protein, determined from the dependence of the initial rate of aggregation measured by AF4 on the initial protein concentration, is equal to 2. The initial stage of aggregation is a relatively slow bimolecular reaction of interaction of two unfolded protein molecules with the formation of a dimer, which is prone to fast aggregation. The stage of protein unfolding proceeds very fast, and the rate-limiting stage of the overall process of aggregation is that of aggregation of unfolded protein molecules. The second order of aggregation with respect to protein was observed, for example, for thermal aggregation of bovine α-chymotrypsinogen A type II (20 mM sodium citrate, pH 5, at 52.5 °C^20^), tobacco mosaic virus coat protein (50 mM phosphate buffer, pH 8.0, at 42 °C and 52 °C^45^), firefly luciferase (25 mM Tricine, pH 7.5, at 42 °C^46^) and apoglycogen phosphorylase *b* from rabbit skeletal muscles (0.08 M Hepes buffer, pH 6.8, containing 0.1 M NaCl and 5 mM DTT at 37 °C^47^).

When studying the kinetics of thermal aggregation of proteins, one should take into account that a variation in temperature can cause a change in the rate-limiting stage of the overall process of aggregation. Earlier we showed^48^ that the initial rate of heat-induced aggregation of rabbit muscle creatine kinase at 50.6 °C (30 mM Hepes-NaOH buffer, pH 6.8) is proportional to the initial protein concentration, suggesting that the order of aggregation with respect to protein is equal to unity (*n* = 1). The latter indicates that the rate-limiting stage of the overall process of aggregation is that of unfolding of the protein molecule and the unfolding stage is followed by fast aggregation of unfolded protein molecules. However when aggregation was studied at 60 °C, the order of aggregation with respect to protein was equal to 2, suggesting that aggregation of the unfolded protein molecules became the rate-limiting stage.

The kinetics of thermal aggregation of BSA at different temperatures has been studied in our previous work^49^. It was shown that at 65 °C (0.1 M sodium phosphate buffer, pH 7.0) the portion of the aggregated protein determined by AF4 is proportional to the portion of denatured protein determined from differential scanning calorimetry (DSC) data. This result shows that denatured protein is rapidly transforming into an aggregated state. Comparing these data with those obtained at 70 °C in the present work, one may conclude that BSA belongs to the proteins demonstrating a change in the kinetic regime of their aggregation at varying temperature.

Test systems based on thermal aggregation and DTT-induced aggregation of target proteins are widely used for screening of agents possessing anti-aggregation activity. When interpreting the protective effect of these agents, we should know what kinetic regime of the aggregation process is realized^36, 39^. If we use test systems where the rate-limiting stage is that of protein unfolding (the order of aggregation with respect to protein is unity), the agents exerting some action on the stability of the native form of the target protein will eventually affect the initial rate of aggregation. The agents stabilizing protein structure will retard protein aggregation. DTT-induced aggregation of BSA at 45 °C (0.1 M Na-phosphate, pH 7.0) belongs to the test systems of the type under discussion^35^. It was shown that Arg and its derivatives (ArgAd and ArgEE) suppressed DTT-induced aggregation of BSA^21^. Judging from the data on BSA thermostability in the presence of Arg and its derivatives studied by DSC (see Supplementary Information; section S3), the protective effect of these chemical chaperones can be explained by their stabilizing action on BSA structure. Dasgupta and Kishore^50^ studied the action of osmolytes (proline, hydroxyproline, glycine betaine, sarcosine and sorbitol) on thermal aggregation of BSA at 60 °C (pH 7.4). Taking into account the selected temperature of the experiments, one can assume that the observed effects of osmolytes are connected with their influence on protein thermostability. For example, suppression of BSA aggregation by sorbitol is consistent with the ability of the latter to increase thermal stability of this protein^51^.

Test systems where the rate-limiting stage is that of aggregation of the unfolded protein molecules have the advantage in that they allow detecting the direct effect of the agents under study on aggregation of the target protein^36^. For this reason in the present work the effects of Arg and its derivatives on heat-induced aggregation of BSA were studied under the conditions where the rate-limiting stage of the overall aggregation process is that of aggregation of the unfolded protein molecules, namely at 70 °C.

**Figure 8:**
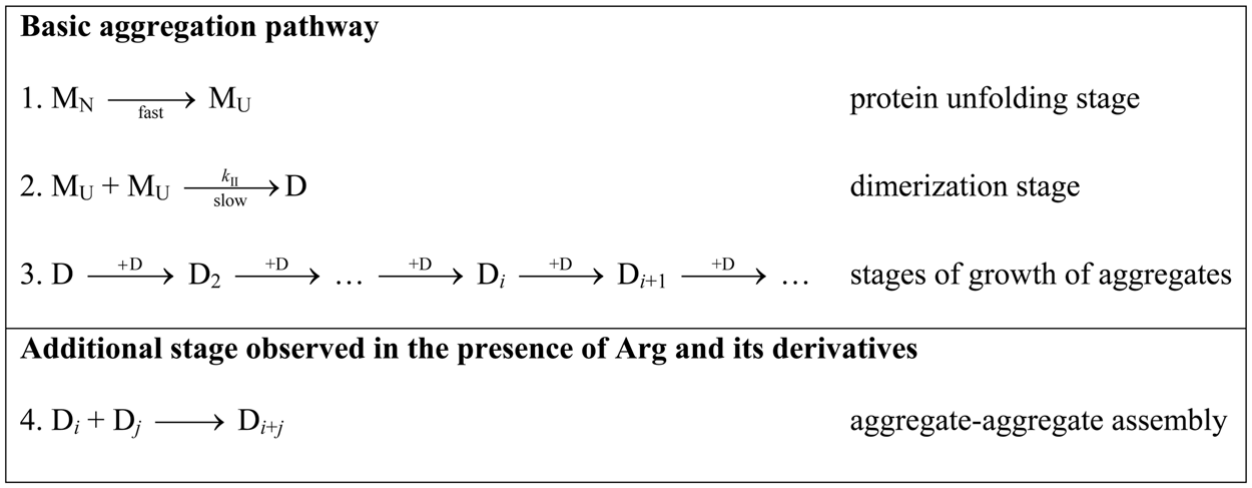
Aggregation pathway for BSA aggregation at 70 °C. M_N_ and M_U_ are the native and unfolded forms of monomeric BSA, respectively. D is a dimer, which is a product of slow dimerization of M_U_. D_2_, D_*i*_, D_*i*+1_, D_*j*_ and D_*i*+*j*_ are aggregates formed by dimers D (subscript is a number of dimers).

On the basis of analysis of the kinetic data for BSA aggregation at 70 °C the following scheme of the aggregation process can be proposed (Fig. 8). The fast stage of protein unfolding (stage 1; M_N_ → M_U_) is followed by the slow dimerization of the unfolded protein molecules with the rate constant of the second order *k*_II_ (stage 2). Further growth of protein aggregates is the result of sequential attachment of dimers D to the existing aggregates: D_*i*_ + D → D_*i*+1_ (stages 3). These stages of the aggregation process can be considered as a basic aggregation pathway (Fig. 8). It should be noted that when the initial protein concentration increases, then acceleration of the dimerization stage follows. However, the shape of *R*_h_ *vs* γ_agg_ plot remains the same at various protein concentrations (Fig. 6). This fact indicates that the aggregation pathway is invariant to varying protein concentration.

The results obtained in the present paper indicate that the larger aggregates of BSA are formed in the presence of Arg, ArgAd and ArgEE. The (d*R*_h_/dγ_agg_) values obtained in the presence of Arg and its derivatives exceed those measured in the absence of chemical chaperones (Fig. 7). To explain this change in the aggregation pathway, one may assume that binding of Arg and its derivatives causes sticking of existing aggregates. Thus, the basic aggregation pathway should be supplemented with the additional stage of aggregate–aggregate assembly D_*i*_ + D_*j*_ → D_*i+j*_ (stage 4 in Fig. 8). The full scheme represented in Fig. 8 illustrates the aggregation pathway for BSA aggregation which is realized in the presence of Arg, ArgAd and ArgEE.

Zeta-potential measurements showed that there was a decrease in the value of zeta-potential of BSA aggregates in the presence of Arg and its derivatives. This decrease in zeta-potential should result in diminishing electrostatic repulsion between colliding particles and stimulating aggregate–aggregate assembling through hydrophobic interactions.

Consider how the relationship between the *k*_II_ and *K*_LS_ values changes in the presence of chemical chaperones. The data represented in Fig. 4A demonstrate that the *k*_II_/*k*_II,0_ ratio decreases, as Arg concentration increases from zero to 400 mM. The values of *K*_LS_/*K*_LS,0_ ratio in this interval of Arg concentrations appear to be higher than the values of *k*_II_/*k*_II,0_ ratio. The relationship between the *k*_II_ and *K*_LS_ values is described by Equation (8). The violation of this relationship in the presence of Arg is due to Arg-induced changes in the ${(I-{I}_{0})}_{\mathrm{lim}}^{0.5}$ value connected with the formation of larger aggregates (Fig. 7).

In the case of ArgAd and ArgEE (Fig. 4B and C) the changes in the *K*_LS_/*K*_LS,0_ values measured at the chemical chaperone concentrations from zero to 100 mM are connected with the increase in both *k*_II_ and ${(I-{I}_{0})}_{\mathrm{lim}}^{0.5}$ values. The higher values of *K*_LS_/*K*_LS,0_ ratio in comparison with *k*_II_/*k*_II,0_ ratio at higher concentrations of the chemical chaperone (100 mM < [ArgAd] < 300 mM and 100 mM < [ArgEE] < 500 mM) are due to the increase in the ${(I-{I}_{0})}_{\mathrm{lim}}^{0.5}$ value in the presence of ArgAd and ArgEE.

Analysis of the data describing the action of Arg and its derivatives on BSA aggregation shows that the dependence of the *k*_II_/*k*_II,0_ ratio on the concentration of ArgAd or ArgEE passes through a maximum, whereas monotonous decrease in the *k*_II_/*k*_II,0_ ratio is observed with increasing Arg concentration. One can assume that the unfolded BSA molecule contains at least two sets of the binding sites for ArgAd (or ArgEE) possessing different affinity to the ligand. The binding of the ligand in the site with high affinity results in the acceleration of the dimerization stage (stage 2 in Fig. 8). When the concentration of ArgAd (or ArgEE) increases, the binding of the ligand in the site with low affinity occurs with a subsequent decrease in the *k*_II_ value. Thus, the depletion of the negative charge by the chemical modification of the carboxylic group in Arg molecule provides the possibility of binding of Arg derivatives in the binding sites greatly differing in their affinity to the ligand.

The dependence of (d*R*_h_/dγ_agg_)/(d*R*_h_/dγ_agg_)_0_ on Arg concentration passes through a maximum (Fig. 7D). This means that the ability of Arg to promote aggregate–aggregate assembly is weakened at high Arg concentrations.

When interpreting the effects of Arg and its derivatives on the rate of BSA aggregation at 70 °C, we should take into account the changes in the properties of the medium created by the addition of chemical chaperones. Arginine molecule exists as a monovalent cation at pH 7.0, whereas molecules of ArgAd and ArgEE are divalent cations (Cl^−^ is a counter-ion). The results of the experiments on the effect of ionic strength on BSA aggregation (Supplementary Information; section S2) showed that the initial rate of aggregation remained unchanged in the interval of NaCl concentrations from 0 to 0.5 M (this interval of NaCl concentrations corresponds to the interval of the values of ionic strength from 0.22 to 0.72; the ionic strength of 0.1 M Na-phosphate buffer, pH 7.0, is assumed to be equal to 0.22). This means that at [Arg] > 500 mM, [ArgAd] > 170 mM and [ArgEE] > 170 mM the changes in the *K*_LS_ value with increasing concentration of the additive can be partially due to the effect of ionic strength on the rate of BSA aggregation.

As it can be seen from Fig. 4, the suppressing effect of Arg and its derivatives is weakened at high concentrations of the additives (higher than 500 mM). One may assume that these chemical chaperones act as crowding agents when they are added in high concentrations. It is well known that crowding stimulates protein aggregation^52–58^. Evidently, crowding created by high concentrations of the chemical chaperone will interfere with the anti-aggregation action of Arg and its derivatives. Since the effect of a crowding agent is determined by its size^59^, one can expect that clusterization of Arg, ArgAd and ArgEE should enhance the crowding ability of these chemical chaperones^60^. DLS can be used to demonstrate the formation of clusters of Arg and its derivatives (see Supplementary Information; section S4).

## Conclusions

When data on the kinetics of protein aggregation obtained by DLS, which allows determining the size of protein aggregates (*R*_h_), are supplemented with the direct measurements of the amount of the aggregated protein (γ_agg_), the construction of *R*_h_ *vs* γ_agg_ plots may provide valuable information on the aggregation pathway. The analysis of the shape of these plots allows us to judge whether the aggregation pathway remains unchanged when the environmental conditions are varied or when the agents affecting protein aggregation are added. With the test system based on thermal aggregation of BSA it has been demonstrated that the analysis of the shape of *R*_h_ *vs* γ_agg_ plot allows us to prove the existence of a stage of aggregate–aggregate assembly in the aggregation pathway, which becomes discernible in the presence of Arg and its derivatives.

## Materials and Methods

### Materials

BSA (catalogue no. A7638, 99+% of purity), L-arginine monohydrochloride (Arg), L-arginine amide hydrochloride (ArgEE) and L-arginine ethyl ester hydrochloride (ArgEE) were purchased from Sigma–Aldrich and used without further purification. All solutions for the experiments were prepared using deionized water obtained with Easy-Pure II RF system (Barnstead, USA). BSA samples were prepared by dissolving solid BSA in 0.1 M phosphate buffer solutions at pH 7.0. All the experiments were performed with freshly prepared solutions of BSA. BSA concentration was determined spectrophotometrically at 280 nm using the absorption coefficient ${A}_{{\rm{cm}}}^{1 \% }$ of 6.58^61^.

### Asymmetric flow field-flow fractionation with on-line multi-angle light scattering (MALS), ultraviolet (UV) and refractive index (RI) detectors

The Eclipse 3 separation system (Wyatt Technology Corporation, USA) based on an Agilent HPLC pump (Agilent Technologies, USA) was used for AF4 experiments. BSA sample in 0.1 M Na-phosphate buffer, pH 7.0, preheated at 70 °C and cooled to room temperature 23 °C, was injected in the separation channel by Agilent autoinjection system (Agilent Technologies, USA). A 21.4 cm channel with a 350-μm channel spacer and ultrafiltration membrane made of regenerated cellulose with a 10-kDa molecular weight cut off (Wyatt Technology Corporation, USA) were used. The flow system was sequentially connected to UV detector (Agilent Technologies, USA), MALS detector (DAWN HELEOS II, Wyatt Technology Corporation, USA) and RI detector (Optilab T-rEX, Wyatt Technology Corporation, USA). The elution was performed with 0.1 M phosphate buffer, pH 7.0, at a flow rate at the channel outlet of 1 mL·min^−1^, 3 mL·min^−1^ cross flow. The data from the detectors were processed in ASTRA software, version 5.3.4 (Wyatt Technology Corporation, USA) to yield the final profiles. The experiment was carried out at room temperature (23 °C). The use of AF4 for the study of the kinetics of BSA aggregation was described in our previous works^21, 35, 49^. The fractogram of intact BSA registered in the interval of the elution time from 12 to 17 min includes three peaks corresponding to the monomeric, dimeric and trimeric forms. In the case of preheated BSA the protein quantified from an area under fractogram in the above-mentioned interval of the elution time was classified as a “non-aggregated protein”.

### Light scattering intensity measurements

For light scattering measurements a commercial instrument Photocor Complex (Photocor Instruments, Inc., USA) was used. A He-Ne laser (Coherent, USA, Model 31–2082, 632.8 nm, 10 mW) was used as a light source. DynaLS software (Alango, Israel) was employed for polydisperse analysis of DLS data. The diffusion coefficient *D* of the particles is directly related to the decay rate τ_c_ of the time-dependent correlation function for the light scattering intensity fluctuations: *D* = 1/2τ_c_ *k* ^2^. In this equation *k* is the wave number of the scattered light, *k = *(4π*n*/λ)sin(θ/2), where *n* is the refractive index of the solvent, λ is the wavelength of the incident light in vacuum and θ is the scattering angle. The mean hydrodynamic radius of the particles, *R*_h_, can then be calculated according to Stokes–Einstein equation: *D* = *k*_B_ *T*/6πη*R*_h_, where *k*_B_ is Boltzmann’s constant, *T* is the absolute temperature and η is the dynamic viscosity of the solvent. The kinetics of heat-induced aggregation of BSA was studied in 0.1 M Na-phosphate buffer, pH 7.0. The buffer was placed in a cylindrical cell with the internal diameter of 6.3 mm and pre-incubated for 5 min at a designed temperature (70 °C). Cells with stopper were used to avoid evaporation. To study the effects of Arg, ArgEE and ArgAd on BSA aggregation, the agents were added to a preheated solution of BSA. When studying the kinetics of aggregation of BSA, the scattering light was collected at a 90° scattering angle. The aggregation kinetics was followed by an increase in the intensity of scattered light (*I*) in time.

The values of the refractive index and dynamic viscosity of Arg, ArgAd and ArgEE solutions, which were used for calculation of the *R*_h_ values from DLS data, are given in Table S1 (see Supplementary Information; section S5).

### Zeta Potential Measurements

Zeta potential of BSA preparations (1 mg·mL^−1^) preheated for 15 min at 70 °C in the absence and in the presence of Arg or its derivatives (200 mM) was measured using Photocor Compact-Z instrument (Photocor Instruments, Inc., USA). Laser with wavelength 654 nm was used as a light source. The measurements were conducted at electrical field voltage 5 V/cm and 20 °C in cylindrical glass vials with disposable Au electrodes. The distance between electrodes was 0.4 cm. The scattered light was collected at a 20° angle.

### Calculations

OriginPro 8.0 SR0 software (OriginLab Corporation, USA) was used for the calculations. To characterize the degree of agreement between the experimental data and calculated values, we used the coefficient of determination (*R* ^2^)^62^.

### Data availability statement

All data generated or analysed during this study are included in this published article (and its Supplementary Information file).

## Electronic supplementary material

Supplementary Information
