# Supplementary material for: A change in the aggregation pathway of bovine serum albumin in the presence of arginine and its derivatives
Source: Sci Rep. 2017 Jun 21;7:3984. doi: 10.1038/s41598-017-04409-x (PMC5479853; doi:10.1038/s41598-017-04409-x)
Supplement: Supplementary file 1 — Supplementary Information [file 41598_2017_4409_MOESM1_ESM.pdf]

Supplementary Information

A change in the aggregation pathway of bovine serum albumin in the presence of arginine and its derivatives

Vera A. Borzova[1], Kira A. Markossian[1], Sergey Yu. Kleymenov[2] & Boris I. Kurganov[1,*]

[1]Federal State Institution "Federal Research Centre "Fundamentals of Biotechnology" of the Russian Academy of Sciences", Leninsky pr. 33, Moscow, 119071, Russia. [2]Kol'tsov Institute of Developmental Biology, Russian Academy of Sciences, 26 Vavilova str, Moscow, 119991, Russia.

*Correspondence and requests for materials should be addressed to B.I.K. (email: kurganov@inbi.ras.ru)

S1. Effect of argininamide (ArgAd) and arginine ethyl ester (ArgEE) on thermal aggregation of bovine serum albumin (BSA)

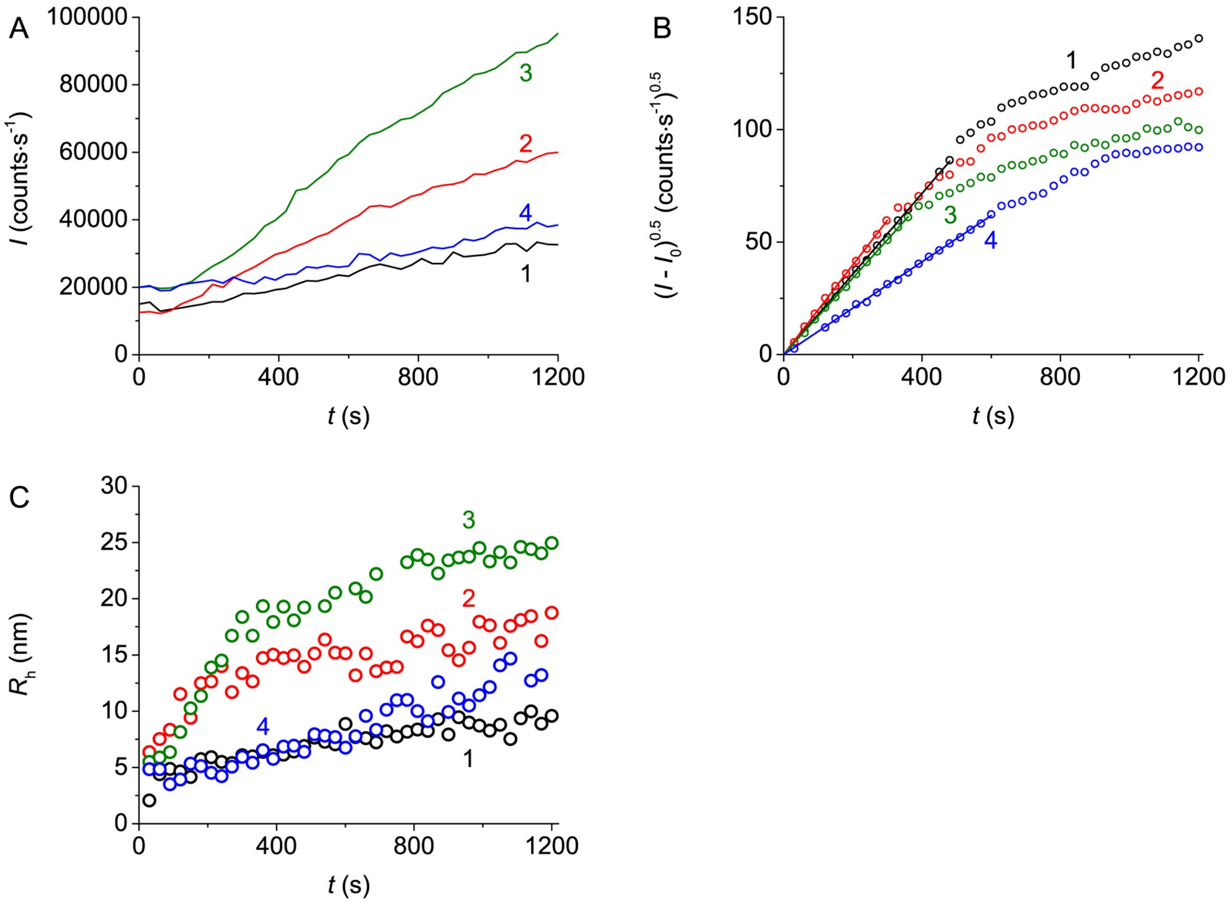

Figure S1. Effect of ArgAd on the kinetics of aggregation of BSA (1 mg·mL^{-1}) registered by dynamic light scattering (DLS) (0.1 M Na-phosphate buffer, pH 7.0, 70 °C). (A) The dependences of the light scattering intensity (I) on time obtained at the following concentrations of ArgAd: 0 (1), 50 (2), 200 (3) and 500 mM (4). (B) The kinetic curves represented in the coordinates $\{(I - I_0)^{0.5}; t\}$. Concentrations of ArgAd: 0 (1), 50 (2), 200 (3) and 500 mM (4). (C) The dependences of the hydrodynamic radius (R_h) of protein aggregates on time obtained at the following concentrations of ArgAd: 0 (1), 50 (2), 200 (3) and 700 mM (4).

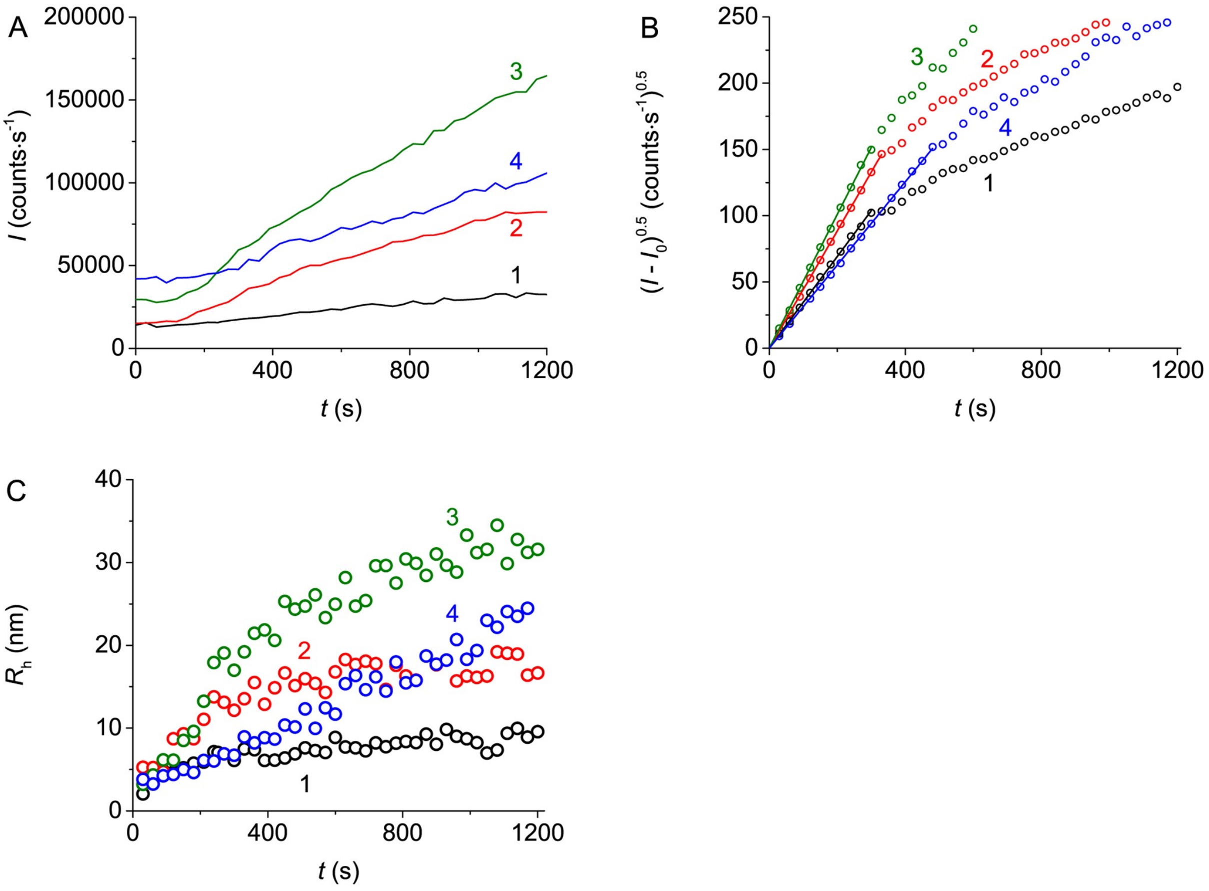

Figure S2. Effect of ArgEE on the kinetics of aggregation of BSA (1 mg·mL^{-1}) registered by DLS (0.1 M Na-phosphate buffer, pH 7.0, 70 °C). (A) The dependences of the light scattering intensity (I) on time obtained at the following concentrations of ArgEE: 0 (1), 50 (2), 200 (3) and 500 mM (4). (B) The kinetic curves represented in the coordinates $\{(I - I_0)^{0.5}; t\}$. Concentrations of ArgEE: 0 (1), 50 (2), 200 (3) and 500 mM (4). (C) The dependences of the hydrodynamic radius (R_h) of protein aggregates on time obtained at the following concentrations of ArgEE: 0 (1), 50 (2), 200 (3) and 1500 mM (4).

S2. Effect of ionic strength on the kinetics of BSA aggregation

To characterize the effect of ionic strength on the rate of BSA aggregation at 70 °C, we studied the kinetics of BSA aggregation at different concentrations of NaCl using DLS. On the basis of the analysis of the initial parts of the kinetic curves (Fig. S3A) the values of the $K_{LS}/K_{LS,0}$ ratio as a function of NaCl concentration were calculated (Fig. S3B). There are no changes in the $K_{LS}/K_{LS,0}$ ratio in the interval of NaCl concentration from 0 to 0.5 M. Further increase in the NaCl concentration results in the diminishing of the $K_{LS}/K_{LS,0}$ ratio. At high concentrations of NaCl (4 M) the $K_{LS}/K_{LS,0}$ ratio markedly exceeds unity. Fig. S3C shows the change in the hydrodynamic radius (R_h) of protein aggregates in the course of BSA aggregation. As it can be seen from this Figure, there are no marked differences between the dependences of R_h on time obtained at different concentrations of NaCl.

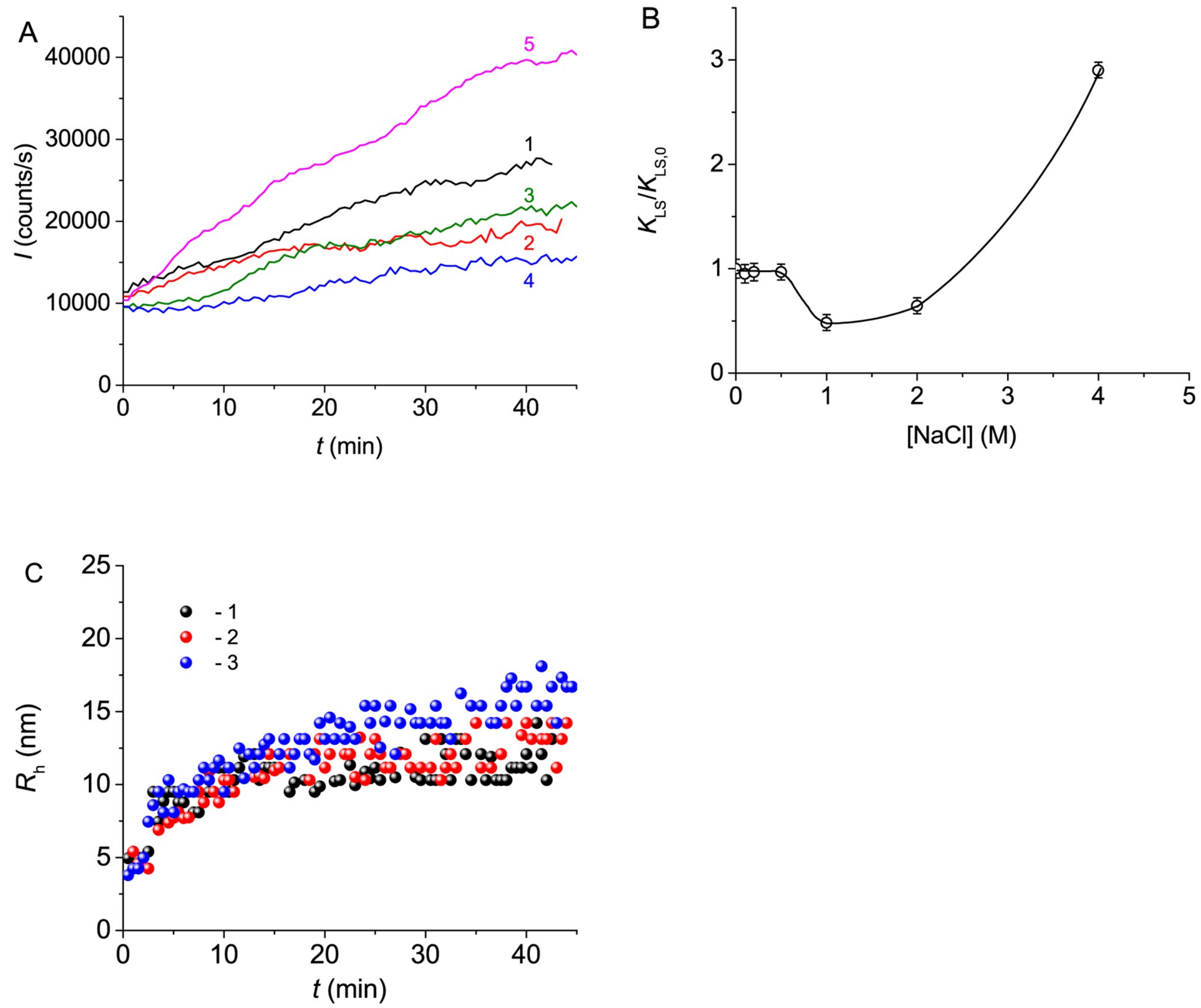

Figure S3. Effect of ionic strength on the kinetics of aggregation of BSA (1 mg·mL^{-1}) at 70 °C (0.1 M Na-phosphate, pH 7.0). (A) The dependences of the light scattering intensity (I) on time obtained at the following concentrations of NaCl: 0 (1), 0.1 (2), 0.5 (3), 1 (4) and 4 M (5). (B) The $K_{LS}/K_{LS,0}$ ratio represented as a function of NaCl concentration. The K_{LS} values were calculated from equation (5). $K_{LS,0}$ is the K_{LS} value in the absence of NaCl. (C) The dependences of the hydrodynamic radius (R_h) of protein aggregates obtained at the following concentrations of NaCl: 0 (1), 0.5 (2) and 4 M (3).

S3. Effects of arginine (Arg) and its derivatives on thermostability of BSA studied by differential scanning calorimetry (DSC)

Thermal denaturation of BSA in 0.1 M sodium phosphate buffer, pH 7.0, was studied by DSC using the adiabatic scanning microcalorimeter DASM-4M (Institute of Biological Instruments, Russian Academy of Sciences, Pushchino, Russia) with 0.47 ml capillary platinum cells. All measurements were carried out at the rate of heating of 1 °C·min^{-1} using the temperature range from 42 °C to 70 °C and constant pressure of 2.2 atm. The dependences of the heat power on temperature were calculated using the program Origin software (MicroCal, Inc., USA). The capillary construction of calorimetric cells prevents the artifacts caused by protein precipitation, which are often observed in batch calorimetric cells as exothermic peaks. All measurements were repeated three times for each sample.

To characterize the effect of Arg and its derivatives (ArgAd and ArgEE) on thermostability of BSA, DSC measurements were carried out in the absence and in the presence of the agents under study. Fig. S4 shows DSC profiles obtained at various concentrations of Arg (A), ArgAd (B) and ArgEE (C). The position of maximum on DSC profile (T_{max}) for original preparation of BSA was found to be 58.5 ± 0.1 °C. The T_{max} value can be considered as a measure of protein thermostability. The position of T_{max} is shifted towards higher temperatures for Arg, ArgAd and ArgEE. Thus, Arg and its derivatives reveal stabilizing effect. It is of interest that low concentrations of ArgAd and ArgEE (70 and 180 mM, respectively) have a maximum stabilizing effect (Fig. S4B and S4C). However, the increase in the concentration of ArgAd and ArgEE results in the diminishing of the stabilizing effect. To explain the change in the character of action of ArgAd and ArgEE on BSA thermostability with varying concentration of the derivative, one can assume that the native BSA molecule also contains non-equivalent binding sites for ArgAd (or ArgEE) possessing different affinity to the ligand. The binding of the ligand in the site with high affinity at relatively low ligand concentrations results in the stabilization of

the BSA molecule. As the concentration of ArgAd (or ArgEE) increases, the binding of the ligand in the site with low affinity occurs with a consequent decrease in the overall stabilizing effect of the Arg derivative.

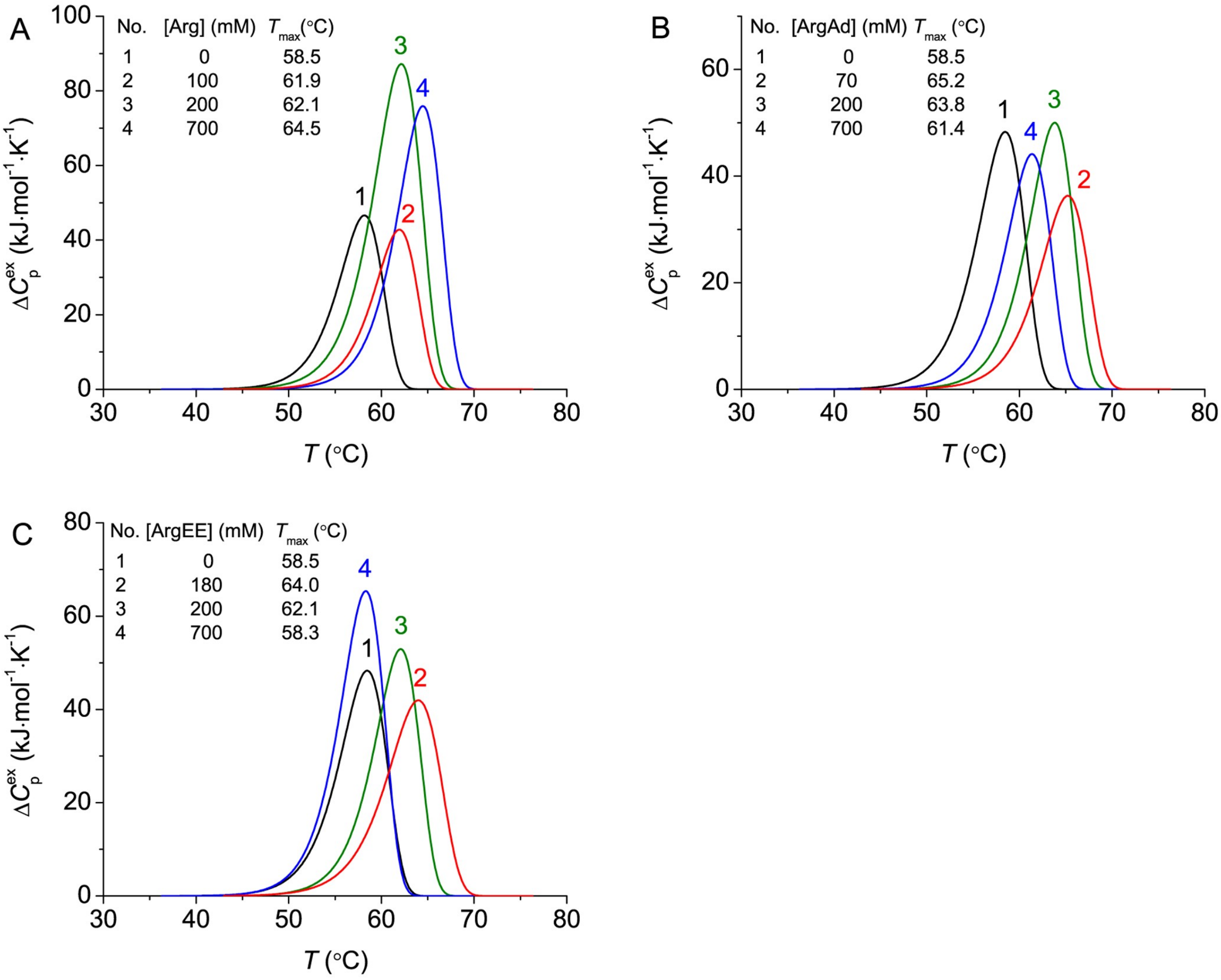

Figure S4. Effects of Arg, ArgAd and ArgEE on thermostability of BSA (1 mg·mL^{-1}). The dependences of excess heat capacity on temperature obtained at various concentrations of Arg (A), ArgAd (B) and ArgEE (C).

S4. Clusterization of Arg and its derivatives

DLS gives the direct evidence for formation of clusters in the solutions of Arg and its derivatives. Fig. S5 shows the distributions of the particles by size in 700 mM Arg (A), 700 mM ArgAd (B) and 500 mM ArgEE (C) at 70 °C (0.1 M Na-phosphate buffer, pH 7.0). As it can be seen, the clusters of Arg and its derivatives can reach rather large sizes (hundreds of nm).

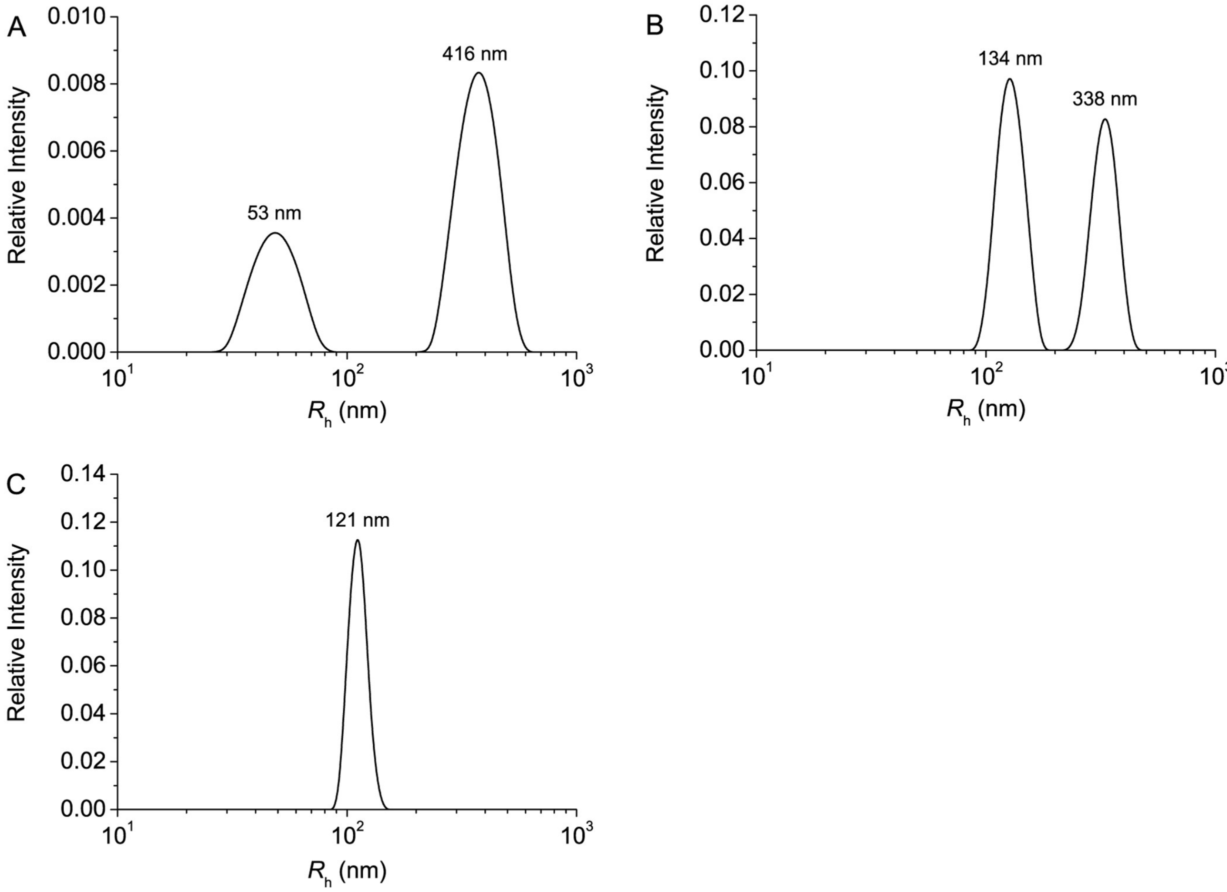

Figure S5. Clusterization of Arg, ArgAd and ArgEE in 0.1 M Na-phosphate buffer, pH 7.0, at 70 °C. Particle size distribution for 700 mM Arg (A), 700 mM ArgAd (B) and 500 mM ArgEE (C).

S5. Determination of refractive index, density and dynamic viscosity

The values of the refractive index of Arg, ArgAd and ArgEE solutions at different concentrations (0.1 M Na-phosphate buffer, pH 7.0) were determined in ABBEMAT 500 refractometer (Anton Paar, Austria) at 70 °C. Density of Arg, ArgAd and ArgEE solutions were determined in density meter DMA 4500 (Anton Paar, Austria). Dynamic viscosities of the solutions were determined in automatic microviscometer (Anton Paar, Austria) in system 1.6/1.500 mm at 70 °C. The obtained values of the refractive index, density and dynamic viscosity of Arg, ArgAd and ArgEE solutions are given in Table S1. The values of refractive index and dynamic viscosity of Arg, ArgAd and ArgEE solutions were used for the determination of the values of the hydrodynamic radius (R_h) of protein aggregates in the DLS measurements.

Table S1. The values of refractive index (*n*), density (ρ) and dynamic viscosity (η) of solutions of Arg, ArgAd and ArgEE at 70 °C (0.1 M Na-phosphate buffer, pH 7.0).

Concentration (mM)	*n*	ρ (g·cm^{-3})	η (mPa·s)
		Arg	
0	1.32747 ± 0.00002	0.99151 ± 0.00005	0.4214 ± 0.0002
50	1.32935 ± 0.00002	0.99914 ± 0.00005	0.4339 ± 0.0002
100	1.33132 ± 0.00002	1.00074 ± 0.00005	0.4437 ± 0.0002
200	1.33488 ± 0.00002	1.00464 ± 0.00005	0.4613 ± 0.0002
350	1.34206 ± 0.00002	1.00870 ± 0.00005	0.5043 ± 0.0002
400	1.34421 ± 0.00002	1.01030 ± 0.00005	0.5160 ± 0.0002
500	1.34874 ± 0.00002	1.02187 ± 0.00005	0.5424 ± 0.0002
700	1.35666 ± 0.00002	1.05755 ± 0.00005	0.7134 ± 0.0002
1000	1.37031 ± 0.00002	1.07623 ± 0.00005	0.9723 ± 0.0002
		ArgAd	
0	1.32747 ± 0.00002	0.99151 ± 0.00005	0.4214 ± 0.0002
50	1.32935 ± 0.00002	0.99914 ± 0.00005	0.4339 ± 0.0002
75	1.33025 ± 0.00002	0.99993 ± 0.00005	0.4377 ± 0.0002
100	1.33132 ± 0.00002	1.00074 ± 0.00005	0.4437 ± 0.0002
150	1.33347 ± 0.00002	1.00231 ± 0.00005	0.4558 ± 0.0002
200	1.33488 ± 0.00002	1.00464 ± 0.00005	0.4613 ± 0.0002
300	1.33992 ± 0.00002	1.00710 ± 0.00005	0.4920 ± 0.0002
500	1.34874 ± 0.00002	1.02187 ± 0.00005	0.5424 ± 0.0002
700	1.35666 ± 0.00002	1.05755 ± 0.00005	0.7134 ± 0.0002
1000	1.37031 ± 0.00002	1.07623 ± 0.00005	0.9723 ± 0.0002
		ArgEE	
0	1.32747 ± 0.00002	1.00982 ± 0.00005	0.4214 ± 0.0002
50	1.32883 ± 0.00002	1.01002 ± 0.00005	0.4393 ± 0.0002
100	1.33234 ± 0.00002	1.01028 ± 0.00005	0.4629 ± 0.0002
200	1.33787 ± 0.00002	1.01096 ± 0.00005	0.5064 ± 0.0002
300	1.34426 ± 0.00002	1.01200 ± 0.00005	0.5498 ± 0.0002
500	1.35589 ± 0.00002	1.01591 ± 0.00005	0.6364 ± 0.0002
700	1.36810 ± 0.00002	1.02479 ± 0.00005	0.7238 ± 0.0002
1000	1.38597 ± 0.00002	1.06323 ± 0.00005	0.8543 ± 0.0002
1500	1.41595 ± 0.00002	1.43077 ± 0.00005	1.0719 ± 0.0002
